# Influence of Social Adversity on Perceived Health Status and Depressive Symptoms among Portuguese Older People

**DOI:** 10.3390/ijerph19116355

**Published:** 2022-05-24

**Authors:** Joana Sampaio, Ana Henriques, Elisabete Ramos, Isabel Dias, Alexandra Lopes, Sílvia Fraga

**Affiliations:** 1 Laboratório para a Investigação Integrativa e Translacional em Saúde Populacional (ITR), Rua das Taipas 135, 4050-600 Porto, Portugal; joana-sampaio@live.com.pt (J.S.); ana.henriques@ispup.up.pt (A.H.); eliramos@med.up.pt (E.R.); 2 EPIUnit, Instituto de Saúde Pública da Universidade do Porto, Rua das Taipas 135, 4050-600 Porto, Portugal; 3 Departamento de Ciências da Saúde Pública e Forenses e Educação Médica, Faculdade de Medicina, Universidade do Porto, Alameda Prof. Hernâni Monteiro, 4200-319 Porto, Portugal; 4 Instituto de Sociologia, Faculdade de Letras da Universidade do Porto, Via Panorâmica, s/n, 4150-564 Porto, Portugal; mdias@letras.up.pt (I.D.); aslopes@letras.up.pt (A.L.)

**Keywords:** aging, abuse, poverty, food insecurity, old-age vulnerabilities

## Abstract

This study aims to investigate how exposure to poverty, food insecurity, and abuse at older ages relates to health outcomes. A questionnaire collecting data on sociodemographic and economic characteristics, health status, depressive symptoms, food insecurity, and abuse was administered to a sample of 677 older adults. Logistic regression was used to quantify the association of poverty, food insecurity, and abuse with perceived health status and depressive symptoms. If the older person only reported experiences of abuse, it was more likely to report the presence of depressive symptoms, even after adjustment for covariates. If it was only reported the experience of food insecurity, it was more likely to report a worse health status. Older people exposed to at least two factors of vulnerability were significantly more likely to report (very) poor perceived health status (OR: 7.11, 95% CI: 2.77–18.25) and the presence of relevant depressive symptoms (OR: 4.34; 95% CI: 2.04–9.22). Thus, the combined effect of vulnerabilities was significantly associated with worse health among older people. Public health policies to mitigate these adverse exposures should be developed to promote health and well-being in this population.

## 1. Introduction

Population aging and the increasing number of older people are a considerable challenge for societies [1]. A wide range of old-age vulnerabilities, such as lack of health-related knowledge and awareness, specific nutritional and physical needs, psychosocial concerns (depression or other neuropsychiatric pathologies) and mistreatment, as well as financial constraints, have a significant impact on the quality of life of older people [2].

Social adversity comprises unfavorable environmental conditions that are related to poor physical health. It includes not only material deprivation but also psychosocial factors, such as abuse experiences, that may accumulate over the life course. In addition, old age vulnerabilities can be exogenous/external events, related to the social and physical environments in which the older people live, or, more frequently, endogenous/internal events, considering older people’s health, employment, and social support issues [3,4,5]. The distribution of vulnerabilities in old age is very unequal because there are events and conditions individuals have no control over (e.g., the global spread of diseases, widowhood, physical and/or cognitive dependence, abuse, and violence). Furthermore, there are dimensions of vulnerability that are socially constructed and developed throughout life (e.g., long-term unemployment, precarious professional trajectories, retirement), greatly influencing the way individuals manage the specific challenges of old age [6].

In many countries, the social protection systems do not assure adequate income security until the end of individuals’ lives, when the expenses tend to increase, in part due to health needs, which makes older people particularly vulnerable to economic insecurity [7]. Individuals are forced to live on limited budgets that may contribute to worse health status (deteriorating physical and mental health and functional abilities) [8] and self-perception of wellbeing [9], as well as an increased risk of chronic diseases and other numerous deleterious health consequences [10].

Comorbidities and frailty, that arise with increasing age [11], are linked with rising risks of the onset of ill health and disability [5,8,12]. Studies showed that in general, older age, being women, lower education, lower economic status and not living with a partner are social adversity factors that were associated with worse health and quality of life [13,14,15,16,17,18].

From the economic instability, inadequate food intake may arise and lead to food insecurity [19], which disproportionately affects individuals living in poverty [20] and could worsen the adverse health consequences, being mental health one of the most affected dimensions [10]. Above all, proper nutrition among this age group is imperative because specific nutritional needs and diets are needed to manage older adults’ health conditions [21].

Some life experiences occurring among older people are also likely to be related to or a sign of an abuse experience, such as changes in the financial situations, changes in behavior or the regular social activity, as well as being left alone for long periods, not having proper clothing or food in the house and the presence of injuries [22]. Elder abuse is a destructive behavior directed at an older person that occurs in a context of trust and it may be psychological, physical, financial, or sexual abuse or neglect [23]. This experience can have particularly negative effects on health in this population, including longer periods of convalescence [23], due to a diminished ability to defend themselves or to escape from the abusive situation, when compared with other population groups [24].

The underlying rationale is that the more difficulties an individual accumulates, the more vulnerable she or he will be [25]. Thus, in Portugal, a country hit by a recent financial crisis, would be expected to observe the co-occurrence of old-age vulnerabilities and it is expected that the accumulation of vulnerabilities will worsen the health consequences.

Therefore, we hypothesized that there might exist a higher risk of worse health outcomes if individuals have a combined exposure to vulnerabilities. The current study aims to investigate how exposure to poverty, food insecurity, and abuse is related to health outcomes, namely self-perceived health status and reported depressive symptoms among older adults.

## 2. Materials and Methods

### 2.1. Study Design and Participants

As part of a Portuguese population-based cohort study—the EPIPorto cohort—of urban dwellers, men and women, previously assembled using random digit dialing [26], a study wave under the acronym HARMED: Socioeconomic and Health Determinants of Elder Abuse was conducted. Between April and October of 2017, all members of the cohort that were 60 years or more were contacted to participate in the HARMED project [27]. A letter explaining the HARMED study was sent by postal mail to all the eligible participants. Individuals were contacted by telephone and those who were willing to take part in the project were scheduled for a face-to-face interview in the Department of Public Health Sciences and Forensics and Medical Education. Data were collected by graduated Social and Health Sciences researchers, that received prior training to assure data collection quality and validity, using a structured questionnaire.

Of the 984 subjects that were contacted, 43 were deceased and 244 refused to participate in the evaluation. Of the 697 individuals who accepted to participate, 20 were excluded because of significant cognitive impairment (Mini-Mental State Examination—MMSE—score < 24, as recommended in previous studies to ensure the validity of self-reported questionnaires in population studies [28]). The final sample included 677 participants.

The local ethics committee (Centro Hospitalar Universitário São João) approved the study protocol (CES-320/2016). Written informed consent was provided and signed by participants. The World Health Organization (WHO) ethical and safety guidelines for the conduct of research on violence against women were followed [29].

### 2.2. Measures

Household monthly income was assessed through one question “What is the average monthly income of your household (Euro), after the mandatory discounts for contributions and taxes?”. Participants also reported their self-perception regarding household monthly income adequacy, then dichotomized into sufficient (that corresponds to ‘allows to live comfortably’ and ‘allows to make ends meet’) and insufficient (that collapses the ‘it is hard to make ends meet’ and ‘it is very hard to make ends meet’ categories), according to the variable distribution.

According to *Instituto Nacional de Estatística* (INE), the at-risk-of-poverty rate is the share of people with an equivalized disposable income below the at-risk-of-poverty threshold, which is set at 60% of the national median equivalized annual disposable income after social transfers (€9351) [30]. The monthly equivalized disposable income was converted to annual income and then was created a dichotomized variable for risk of poverty, with two categories: ‘no’ (when above the at-risk-of-poverty threshold) and ‘yes’ (when below the at-risk-of-poverty threshold), based on the INE guidelines [30].

Household food security was assessed through the previously validated US Household Food Security Survey Module: Six-Item Short Form, which comprises six questions and was translated to the Portuguese language by the EPIUnit research team (Cronbach α 0.748) [31]. Household food security status was classified as high or marginal, low, and very low. Since the proportion of participants in the two food insecure levels (‘low food security’ and ‘very low food security’) was very low, we decided to merge them into one category only. Household food security status was dichotomized for analysis as ‘food-secure’ vs. ‘food-insecure’.

Exposure to abuse was self-reported considering 52 items on psychological, physical, financial, and sexual violence and neglect, based on the Revised Conflict Tactic Scales [32] and the UK survey of elder abuse and neglect [33]. The several items of each type of abuse were collapsed to define a binary outcome variable for each type of abuse. To assess last year’s abuse, we considered ‘yes’ if they reported any abuse in the past 12 months and ‘no’ if they only reported abuse in the previous years or none ever.

Participants were also asked in one question about their self-perception of health, rating their health status by choosing one of five categories: ‘very good, ‘good’, ‘reasonable’, ‘poor’ and ‘very poor’. These categories were merged into ‘very good/good’, ‘reasonable’ and ‘poor/very poor’ and, for the logistic regression, dichotomized into ‘very good/good or reasonable’ and ‘poor/very poor’. Depressive symptoms were assessed with the translated and validated Portuguese version of the short-form of the Geriatric Depression Scale [34], a 15 items scale. The total score ranges from 0 to 15, a total score higher than five suggests the presence of depressive symptoms.

Sociodemographic and economic variables were assessed, including age, gender, marital status, education, occupational position, housing (own, rented, familiar, social, rent-free houses), and financial support (working, social/sick-leave/other pensions benefits, and other financial support that included financial transfers of (ex-)partner/spouse, children or other family members, other type or no financial support). Education was recoded into three levels (high: >9 years; intermediate: 5–9 years; low: <5 years). The occupational position was grouped into three categories: high (upper white collar), intermediate (lower white-collar), and low (blue-collar) [35,36]. Retired participants were classified considering their main occupation before retirement. Social support was assessed with the Multidimensional Scale of Perceived Social Support [37], previously translated to Portuguese (Cronbach α 0.88) [38]. It contains 12 items graded from 1–7 (totally disagree-totally agree). High scores correspond to high social support. Information on chronic diseases encompassing any disease that requires regular medical care was self-reported by participants.

### 2.3. Statistical Analysis

Descriptive statistics, as mean and standard deviation (SD) or counting and proportions, whenever applicable, were computed to describe participants. The Chi-square test was used to compare proportions. Crude and adjusted Odds Ratio (OR) and 95% confidence intervals (95% CI) were calculated using logistic regression. Covariates were selected a priori, considering the literature review and those which were known to be moderators or confounders in the effect of vulnerabilities on perceived health status and depression, such as age, gender, marital status, education, occupation, chronic diseases, monthly household income, and perceived social support were tested. For each model, we tested the contribution of all covariates separately. However, only the ones who changed the magnitude of the association by, at least, 10% (change in the adjusted OR compared with crude OR), were included in the final models: gender, marital status, and perceived social support. Also, we evaluated the fit of our models and we found a good fit of the data for all the models (Hosmer-Lemeshow *p* > 0.05). Statistical analysis was performed using the PASW Statistics version 23.0 for Windows (SPSS Inc., Chicago, IL, USA). A significant level of *p* < 0.05 was set.

## 3. Results

Most of the participants were women and were married or cohabiting with a partner. Approximately 40% of the participants had high education and occupational position (Table 1). Table 1 shows that the risk of poverty is significantly more prevalent among older participants, those who are not married or cohabiting, and individuals with low education or low occupation. Food insecurity was significantly more prevalent among women and, likewise poverty, among participants who are not married or co-habiting. Still, food insecurity was significantly more common among participants with low education and low occupational position.

The majority of the participants perceived their monthly household income as sufficient (84.4%), had their own house (63.4%), and had social/sick-leave or other pension benefits (77.4%) as their main income source (Table 2). Table 2 also shows that the risk of poverty and food insecurity is significantly more prevalent among participants who perceived their monthly household income as insufficient and who have rented or lived in other types of housing. Additionally, exposure to any type of abuse was significantly more frequent among participants who perceived their monthly household income as insufficient.

Most of the participants reported having high social support (68.4%) and chronic diseases (93.8%) but only 6.6% perceived their health as (very) poor and 27.4% had depressive symptoms (Table 3). Poverty is more frequent among participants with moderate or low social support, who perceived their health as ‘poor or very poor’ (34.1%) and who reported relevant depressive symptoms (18.2%). Food insecurity was significantly more common among participants who perceived their health as poorer (28.9%) and reported relevant depressive symptoms (15.2%). Exposure to any type of abuse was more prevalent among participants who have reported depressive symptoms (37.6%) (Table 3).

As presented in Table 4, after adjustment for gender, marital status, and perceived social support, participants who only reported being exposed to food insecurity were significantly more likely to perceive their health as (very) poor (OR: 5.01; 95% CI: 1.19–21.14), and participants who reported only to be exposed to abuse were significantly more likely to report the presence of depressive symptoms (OR: 2.42; 95% CI: 1.52–3.86). However, a dose-response association was observed between the combined exposure to these old-age vulnerability factors and (very) poor perceived health status (exposed to one vulnerability OR: 2.54; 95% CI: 1.20–5.34; exposed to at least two vulnerabilities OR: 7.11; 95% CI: 2.77–18.25) and self-reported depressive symptoms (exposed to one vulnerability OR: 2.20; 95% CI: 1.46–3.32; exposed to at least two vulnerabilities OR: 4.34; 95% CI: 2.04–9.22) (Table 3).

## 4. Discussion

This study shows that, in a population of older adults, combined exposure to poverty, food insecurity, and abuse is significantly associated with worse health outcomes, participants were more likely to perceive their health as poor and to report the presence of relevant depressive symptoms.

Our findings showed a 6.6% prevalence of food insecurity in elders. This prevalence is substantially lower than the ones observed by previous studies, also conducted among Portuguese older people, which reported prevalence estimates of 19 and 23% [39,40]. The presence of food insecurity has been consistently associated with lower education [41,42,43,44]: most of the participants of the two studies mentioned [39,40] presented lower levels of education (less than 5 years), compared with our sample in which most participants had higher levels of education (more than 9 years). Studies developed in other countries, also with older adults, also presented very different prevalence estimates of food insecurity ranging from 2.8% in Australia to 67% in Mexico [42,45,46,47,48,49]. These different estimates among different countries and cultures are expected since the socioeconomic and demographic conditions of the populations are not the same. In addition, once more, the way of measuring food insecurity in the households was different, which could also influence prevalence estimates. Analyzing the isolated impact of the vulnerabilities, food insecurity seems to have a worse impact on health, as it is associated with both worse perceived health status and the presence of depressive symptoms. Involuntary unemployment, early retirement, poverty, or residing in poorer areas are associated with food insecurity [50] and older people are particularly vulnerable to this economic insecurity due to inadequate social protection systems. Then, individuals are forced to live on limited budgets, in which they do not have enough money to spend on food, which will worsen their health and could lead to malnutrition and other chronic diseases, thereby, affecting physical and psychological health and functional capacity [21]. On the other side, limited resources may also postpone needed medical care, which increases health costs and leads to poorer disease management and health status [51]. The more severe the food insecurity, the more compromised a large spectrum of basic needs at risk will be [51]. Regarding the sociodemographic and economic profile of food-insecure participants, we observed that they had had low education, and occupation, insufficient perception of their household income, reported depressive symptoms, lack of or moderate social support and perceived their health status as poor/very poor. Similar characteristics were previously reported in the study of Fernandes and colleagues [39] regarding gender and education and in other studies [42,49,52] regarding income and health status, which is in line with this study results. These findings alert for the need of public health policies and actions to reduce the impact of food insecurity targeting these vulnerable social groups. It was also previously shown that an environment with food insecurity also triggers abuse [53]. Despite the lack of literature about these relationships, some studies have been developed with mothers and support these findings. A study with women aged 18 years or older showed higher odds of intimate partner abuse among those reporting more severe food insecurity [54]. Similar results were found in the study of Hernandez and colleagues reporting that mothers who suffered from intimate partner abuse had an increased risk of food insecurity two years later [55].

Our results showed that approximately 24% of the participants reported abusive behaviors in the last 12 months. Similar prevalence estimates were reported by Martins and colleagues [56] with 23.5% of respondents suffering some kind of abuse and mistreatment and by Gil and colleagues [57] with a slightly lower prevalence of 12.3%. In our study, we observed that psychological abuse was the most frequent (19.9%) in the last 12 months. Other studies among Portuguese elders showed similar results, with a prevalence of psychological abuse of 21.9% in the Abuse and health among elderly in Europe (ABUEL) study [58,59]. A lower prevalence (6.3%) was reported by Gil and colleagues [57], possibly explained by the participants’ levels of education, since it has been shown that psychological abuse is more frequently reported by higher educated individuals [59,60,61]. This reasoning could help to understand the difference in psychological abuse prevalence between our research and Gil and colleagues’ study [57], once the majority of our sample is highly educated (40.2%), contrary to the other study sample (18.2%). The results of our sensitivity analysis corroborated these findings since we observed that adjusting for education increased the association between exposure to abuse and food insecurity. The higher prevalence of psychological abuse was also found in other European countries, as in Germany, Greece, Italy, Lithuania, Spain, and Sweden [59], endorsing our findings. The association between socioeconomic factors and abuse did not show consistency because abuse may occur and be reported in every socioeconomic stratum [62]. However, if abuse occurs in a context characterized by other vulnerabilities, it will contribute to worsening the wellbeing status of the older person [62].

Self-perceived health is usually used as a reliable indicator of overall health status, morbidity and mortality and seems to have a positive association with sociodemographic characteristics, such as education and income, and a negative relationship with chronic diseases, functional dependence, depression, in older age [63]. Food insecure people, that consequently may be at risk of poverty due to worse economic conditions, tend to report a worse health status [52], which is in line with this study results [39]. Besides poverty and food insecurity, abuse also seems to have a negative association with the perceived health status [22]. Late-life depression is a devastating health problem that is associated with an increased risk of morbidity and suicide and decreased physical, cognitive, and social status, with individuals with higher education, socioeconomic status, and social support being less prone to suffer from it [64]. Food insecurity seems to be associated with an increase the risk of chronic diseases, among which depression is one of them [10,65,66]. Previous literature demonstrates that the causal pathway between food insecurity and psychiatric illnesses is bidirectional [67]. Depression is also considered a risk factor for elder abuse [22] and the association can also be bidirectional. Therefore, once every single old-age vulnerability seems to be related either to perceived health or depressive symptoms, it is possible to assume that the co-occurrence of these vulnerabilities could lead to an increased risk of worse perceived health status and more reported, and possibly severe, depressive symptoms.

Nevertheless, individuals who have higher social support seem to have a lower risk of experiencing these vulnerabilities once social support attenuates the detrimental effects of economic constraints in depression [68]. A previous study also showed that high social support may attenuate social inequalities in health at older ages [69]. Literature, however, suggests that this is probably a very complex relationship between several aspects that may model the impact of life course vulnerabilities on health once it could be moderated by several biopsychosocial factors [70]. Higher educational and occupational, as well as cognitive and non-cognitive physical or leisure time activities, levels and other beneficial societal changes may underlie a decrease in age-associated psychiatric problems prevalence and incidence in high-income countries [71,72]. However, these epidemiological reserve-related factors are interrelated: literacy may be determined either by genetics or by educational experiences, and social and environmental factors; occupational status is related to education and socioeconomic characteristics and inequalities. So, longitudinal life course studies are needed to fully understand the complex mechanism of neuropsychiatric problems development and risk [72].

These results were observed during a time of economic recovery in Portugal which leads us to speculate that, in a time where an economic crisis is emerging after COVID-19, this relationship can worsen after the pandemic, putting older people in a more difficult situation and increasing suffering.

Some strengths and limitations of this study should be acknowledged. Data were obtained from a large population-based urban cohort, located in a high-income country. Likewise, we used widely accepted tools for the assessment of our study variables. We used the MMSE to screen cognitive impairment and we exclude those participants who score < 24, we might, however, be excluding participants with mild and severe cognitive impairment that might be also exposed to abuse. When participants and nonparticipants were compared, statistically significant differences were observed in age and education, being the participants younger and more educated, those who are more prone to participate. More than a third of the participants had a higher level of education and occupational position, suggesting that this population sample might be biased towards a high socioeconomic position. Although the cross-sectional nature of the study does not allow to infer a causal relation, it is clear that an environment with poverty and food insecurity and abuse predicts worse health. Also, we are probably underestimating the effects of poverty as our sample is highly educated.

## 5. Conclusions

Aging leads to frailty and, therefore, puts older people at risk of adverse factors (unfavorable economic context, situations of abuse, as well as others). These adversities will not only negatively impact their quality of life and their autonomy but will also place a heavy burden on resources and health and social care services. The particular needs of older people in later life have to be met not only by their families but also by society. Therefore, actions and policies to support this population should be more oriented through a vulnerability perspective, in which promoting more formal and informal support to this population could contribute to reducing the likelihood of experiencing these problems. So, improving socioeconomic conditions and specific legislation against elder abuse would have the potential to benefit the health and well-being of this population.

## Figures and Tables

**Table 1:** Prevalence of at-risk-of-poverty, food insecurity, and abuse, according to sociodemographic characteristics (N = 677).

	*n* (%)	At-Risk-of-Poverty * (%)		Food Insecurity (%)		Abuse (%)	
		No 553 (81.7)	Yes 76 (12.1)	No 632 (93.4)	Yes 45 (6.6)	No 515 (76.1)	Yes 162 (23.9)
**Gender**							
**Women**	428 (63.2)	86.2	13.8	91.1	8.9	73.8	26.2
**Men**	249 (36.8)	90.8	9.2	97.2	2.8	79.9	20.1
		*p* = 0.108		*p* = 0.004		*p* = 0.090	
**Age (years)**							
**60–69**	306 (45.2)	92.0	8.0	94.1	5.9	77.1	22.9
**70–79**	244 (36.0)	85.6	14.4	93.0	7.0	71.7	28.3
**>79**	127 (18.8)	81.2	18.8	92.1	7.9	81.9	18.1
		*p* = 0.006		*p* = 0.727		*p* = 0.079	
**Marital status**							
**Married/Cohabiting**	418 (61.7)	90.8	9.2	95.2	4.8	76.1	23.9
**Other**	259 (38.3)	82.9	17.1	90.3	9.7	76.1	23.9
		*p* = 0.005		*p* = 0.021		*p* = 1.000	
**Education**							
**High (>9 years)**	272 (40.2)	96.9	3.1	98.9	1.1	72.1	27.9
**Intermediate (5–9 years)**	157 (23.2)	93.8	6.3	94.9	5.1	81.5	18.5
**Low (<5 years)**	247 (36.5)	73.9	26.1	86.2	13.8	76.9	23.1
		*p* < 0.001		*p* < 0.001		*p* = 0.079	
**Occupational position**							
**High (Upper white collar)**	276 (43.6)	98.5	1.5	97.5	2.5	75.4	24.6
**Intermediate (Lower white collar)**	198 (31.3)	84.9	15.1	93.4	6.6	76.3	23.7
**Low (Blue collar)**	159 (25.1)	76.8	23.2	87.4	12.6	79.2	20.8
		*p* < 0.001		*p* < 0.001		*p* = 0.647	

Legend: * the at-risk-of-poverty rate was calculated according to INE guidelines; *p*-value for the Chi-square test.

**Table 2:** Prevalence of at-risk-of-poverty, food insecurity, and abuse, according to economic characteristics (N = 677).

	*n* (%)	At-Risk-of-Poverty * (%)		Food Insecurity (%)		Abuse (%)	
		No 553 (81.7)	Yes 76 (12.1)	No 632 (93.4)	Yes 6.6%	No 553 (81.7)	Yes 76 (12.1)
**Perceived household income adequacy**							
**Sufficient**	570 (84.4)	93.1	6.9	97.5	2.5	77.9	22.1
**Insufficient**	105 (15.6)	58.5	41.5	70.5	29.5	65.7	34.3
		*p* < 0.001		*p* < 0.001		*p* = 0.010	
**Housing**							
**Own house**	429 (63.4)	91.2	8.8	97.0	3.0	76.9	23.1
**Rented house**	182 (26.9)	82.0	18.0	87.4	12.6	75.3	24.7
**Other types of housing ^†^**	66 (9.7)	81.8	18.2	86.4	13.6	72.7	27.3
		*p* = 0.003		*p* < 0.001		*p* = 0.726	
**Financial support ^1^**							
**Working**	98 (14.5)	91.7	8.3	95.9	4.1	73.5	26.5
**Social/sick-leave/other pensions benefits**	524 (77.4)	86.8	13.2	92.6	7.4	76.3	23.7
**Other**	55 (8.1)	91.5	8.5	96.4	3.6	78.2	21.8
		*p* = 0.305		*p* = 0.305		*p* = 0.771	

Legend: * the at-risk-of-poverty rate was calculated according to INE guidelines; *p*-value for the Chi-square test; ^†^ familiar, social, rent-free houses; ^1^ other financial support included financial transfers of (ex-)partner/spouse, children or other familiars, other type or no financial support.

**Table 3:** Prevalence of at-risk-of-poverty, food insecurity, and abuse, according to health-related characteristics (N = 677).

	*n* (%)	At-Risk-of-Poverty * (%)		Food Insecurity (%)		Abuse (%)	
		No 553 (81.7)	Yes 76 (12.1)	No 632 (93.4)	Yes 6.6%	No 553 (81.7)	Yes 76 (12.1)
**Perceived Social Support ^1^**							
**High support**	458 (68.4)	90.6	9.4	96.9	3.1	77.9	22.1
**Moderate/low support**	212 (31.6)	81.7	18.3	85.4	14.6	71.7	28.3
		*p* = 0.003		*p* < 0.001		*p* = 0.096	
**Perceived health status**							
**Good/Very good**	308 (45.5)	93.8	6.2	97.7	2.3	78.2	21.8
**Reasonable**	324 (47.9)	85.3	14.7	92.3	7.7	75.9	24.1
**Poor/Very poor**	45 (6.6)	65.9	34.1	71.1	28.9	62.2	37.8
		*p* < 0.001		*p* < 0.001		*p* = 0.062	
**Presence of depressive symptoms ^2^**							
**No**	472 (72.6)	90.5	9.5	97.0	3.0	81.6	18.4
**Yes**	178 (27.4)	81.8	18.2	84.8	15.2	62.4	37.6
		*p* = 0.005		*p* < 0.001		*p* < 0.001	
**Presence of chronic diseases**							
**No**	42 (6.2)	97.4	2.6	92.9	7.1	81.0	19.0
**Yes**	634 (93.8)	87.3	12.7	93.4	6.6	75.7	24.3
		*p* = 0.103		*p* = 1.000		*p* = 0.559	

Legend: * the at-risk-of-poverty rate was calculated according to INE guidelines; *p*-value for the Chi-square test; ^1^ perceived social support was assessed by the Multidimensional Scale of Perceived Social Support; ^2^ depressive symptoms were assessed with The Geriatric Depression Scale.

**Table 4:** Logistic Regression analysis of the association between exposure to poverty, food insecurity, abuse, and the combined exposure with (very) poor perceived health status and the presence of depressive symptoms (N = 629).

	Perceived Health Status: (Very) Poor				Presence of Depressive Symptoms			
	Unadjusted Model		Adjusted Model ^†^		Unadjusted Model		Adjusted Model ^†^	
	OR	95% CI	OR	95% CI	OR	95% CI	OR	95% CI
**Exposure to vulnerabilities**								
**Never exposed to any vulnerability**	**Ref.**	**-**	**Ref.**	**-**	**Ref.**	**-**	**Ref.**	**-**
**Only exposed to poverty**	3.61	1.23–10.55	2.83	0.95–8.44	1.83	0.91–3.68	1.45	0.69–3.03
**Only exposed to food insecurity**	7.68	1.92–30.61	5.01	1.19–21.14	4.76	1.56–14.55	3.20	0.98–10.39
**Only exposed to abuse**	2.22	0.94–5.27	2.11	0.88–5.06	2.61	1.67–4.07	2.42	1.52–3.86
**Exposed to at least 2 vulnerabilities**	9.38	3.84–22.90	7.12	2.78–18.25	5.71	2.81–11.58	4.34	2.04–9.22
**Combined exposure to vulnerabilities**								
**Never exposed to vulnerabilities**	**Ref.**	**-**	**Ref.**	**-**	**Ref.**	**-**	**Ref.**	**-**
**Exposed to 1** **vulnerability**	2.92	1.41–6.06	2.54	1.20–5.34	2.52	1.70–3.73	2.20	1.46–3.32
**Exposed to at least 2 vulnerabilities**	9.38	3.84–22.90	7.11	2.77–18.25	5.71	2.81–11.58	4.34	2.04–9.22

Legend: Ref.—reference class; CI—confidence interval; ^†^ Adjusted model—unadjusted model further adjusted for gender, marital status, and perceived social support.

## Data Availability

Data were obtained from the EPIPorto cohort (https://ispup.up.pt/en/coorte/epiporto-2/ (accessed on 20 May 2022). Data from this structure can be assessed upon request to the coordination.
